# The impact of sleep quality on asthma incidence in middle-aged and older adults: evidence from a prospective cohort study

**DOI:** 10.3389/fpubh.2025.1646053

**Published:** 2025-07-31

**Authors:** Rujian Yu, Zeping Chen, Lamei Zou

**Affiliations:** ^1^Department of Pulmonary Disease, No.3 Affiliated Hospital of Chengdu University of Traditional Chinese Medicine (West District), Chengdu Pidu District Hospital of Traditional Chinese Medicine, Chengdu, China; ^2^Chengdu University of Traditional Chinese Medicine, Chengdu, China; ^3^Department of Geriatric, No.3 Affiliated Hospital of Chengdu University of Traditional Chinese Medicine (West District), Chengdu Pidu District Hospital of Traditional Chinese Medicine, Chengdu, China

**Keywords:** sleep quality, sleep duration, asthma, English longitudinal study of ageing, middle-aged and older adults

## Abstract

**Background:**

Sleep disorders represent a growing public health burden, while asthma persists as a predominant chronic respiratory condition globally. Although suboptimal sleep quality correlates with diverse adverse health outcomes, its prospective association with asthma incidence in middle-aged and older adults remains inadequately characterized.

**Methods:**

Utilizing data from the English Longitudinal Study of Ageing (ELSA), this cohort study included 4,578 asthma-free participants aged ≥50 years at baseline. Baseline sleep quality was quantified via a validated questionnaire, classifying participants into high, moderate, and low quality strata; nocturnal sleep duration was concurrently assessed. Incident asthma diagnoses over a 10-year follow-up period constituted the primary endpoint. Multivariable Cox proportional hazards regression models estimated hazard ratios (HRs) for asthma risk, adjusting for demographic, lifestyle, and comorbidity covariates.

**Results:**

Among 4,578 participants, 156 incident asthma cases (3.41%) emerged during follow-up. Following comprehensive adjustment, baseline sleep quality demonstrated a significant dose–response relationship with asthma risk (P for trend < 0.001). Relative to the high-quality reference group, moderate- and low-quality groups exhibited 63% (HR = 1.63; 95% CI: 1.09–2.42) and 84% (HR = 1.84; 95% CI: 1.16–2.92) elevations in asthma risk, respectively. No statistically significant association emerged between sleep duration and asthma incidence.

**Conclusion:**

This large-scale prospective cohort study demonstrates that poor sleep quality is an independent risk factor for the development of asthma in middle-aged and older adults, independent of sleep duration. The findings highlight the potential importance of optimizing sleep quality (rather than simply extending sleep duration) in asthma primary prevention. Improving sleep quality may represent a novel intervention target to reduce asthma incidence in this population and provide evidence for public health strategies.

## 1 Introduction

Asthma is a common chronic inflammatory airway disease characterized by reversible airflow limitation, airway hyperresponsiveness, and airway remodeling (1). Globally, asthma represents a significant public health issue, imposing considerable health and economic burdens (2). Between 1990 and 2019, the incidence and mortality rates of asthma have generally declined (3). According to global burden of disease data, the incidence of asthma decreased from 601.20 per 100,000 people to 477.92 per 100,000, and the mortality rate dropped from 8.60 to 5.96 per 100,000 (3). Despite significant advances in asthma treatment over the past decades, a substantial proportion of patients still experience poor asthma control, severely affecting their quality of life (4) and leading to increased healthcare resource consumption. Although asthma’s etiology is complex, involving genetic, environmental, and other factors, identifying modifiable risk factors is crucial for primary prevention.

In recent years, sleep disorders have attracted growing attention due to their widespread nature and their association with various chronic diseases such as cardiovascular and metabolic disorders (5–7). Sleep disturbances, particularly poor sleep quality and inadequate sleep duration, are recognized as significant contributors to numerous health conditions. Crucially, sleep fragmentation—characterized by frequent arousals and disrupted sleep architecture—has been implicated in cardiovascular pathogenesis through mechanisms such as sympathetic activation, endothelial dysfunction, and systemic inflammation (8). Polysomnography (PSG) remains the gold standard for diagnosing sleep disorders, enabling comprehensive assessment of sleep stages, respiratory events, and limb movements (9, 10). Among sleep disorders, obstructive sleep apnea (OSA) is notably prevalent and clinically significant due to its direct impact on nocturnal hypoxia and systemic inflammation—factors that may exacerbate comorbid conditions like asthma. However, the relationship between sleep characteristics—especially sleep quality and sleep duration—and asthma risk remains underexplored, despite a growing body of research. While studies have suggested that poor sleep may exacerbate asthma symptoms and impair asthma control, the relative contributions of sleep quality versus sleep duration in asthma incidence and severity remain a critical gap in the literature.

The relationship between asthma and sleep is complex and bidirectional (11). In individuals with asthma, symptoms such as coughing, wheezing, and shortness of breath are often more prevalent at night, leading to frequent awakenings, difficulty falling asleep, and fragmented sleep (12, 13). Additionally, lung function tends to decrease during the night due to circadian variations in airway tone, increasing the likelihood of nocturnal exacerbations and further deteriorating sleep quality (14, 15). These disturbances can, in turn, impair sleep quality, creating a cycle that exacerbates both asthma symptoms and sleep problems (16). Conversely, poor sleep quality—whether due to sleep deprivation, sleep disorders, or inadequate sleep duration—can elevate inflammatory markers, aggravating bronchial inflammation and asthma symptoms (17–19). This bidirectional relationship suggests that not only does asthma contribute to impaired sleep quality, but poor sleep also contributes to asthma exacerbations, highlighting the need to explore the causal mechanisms that underlie this interaction.

Emerging evidence has investigated the relationship between sleep disturbances and asthma susceptibility and management. A U. S. National Health and Nutrition Examination Survey cross-sectional analysis identified significant associations between asthma risk and comorbid depression and adverse sleep conditions (encompassing poor sleep quality, sleep disorders, and sleep deprivation), with evidence of depressive-sleep interactions potentiating asthma risk (20). Concurrently, empirical studies substantiate linkages between sleep deprivation and multiple chronic diseases, including asthma (21). Prospective cohort designs further demonstrate that chronic insomnia symptoms elevate asthma incidence (22, 23), while suboptimal sleep quality correlates with diminished asthma control and impaired pulmonary function (13, 24, 25). Nevertheless, methodological heterogeneity persists, as certain investigations report null associations between asthma and poor sleep quality (26). Notably, large-scale cross-sectional analyses examining sleep deprivation-asthma relationships yield divergent results (26–35): while several studies report positive correlations (28, 29, 31, 33), others document non-significant associations (27, 36).

Despite these advances, prospective cohort studies examining the long-term effects of sleep quality and sleep duration on asthma risk are relatively limited, particularly in older adults who are at high risk for both sleep disorders and asthma. This cohort, being at the intersection of aging and chronic disease, represents a crucial population for targeted asthma prevention strategies, as delayed management often leads to accelerated lung function decline and higher mortality.

Therefore, a key issue that needs to be addressed in current research is the distinction between the independent contributions of sleep quality and sleep duration to the risk of new-onset asthma in middle-aged and older adults. This study utilizes data from the English Longitudinal Study of Ageing (ELSA) cohort to explore the associations between baseline sleep quality, sleep duration, and the 10-year risk of new-onset asthma in individuals aged 50 and older, using Cox proportional hazards regression models. The findings aim to provide new scientific evidence for primary prevention strategies for asthma.

## 2 Methods

### 2.1 Study design and population

**Figure 1:**
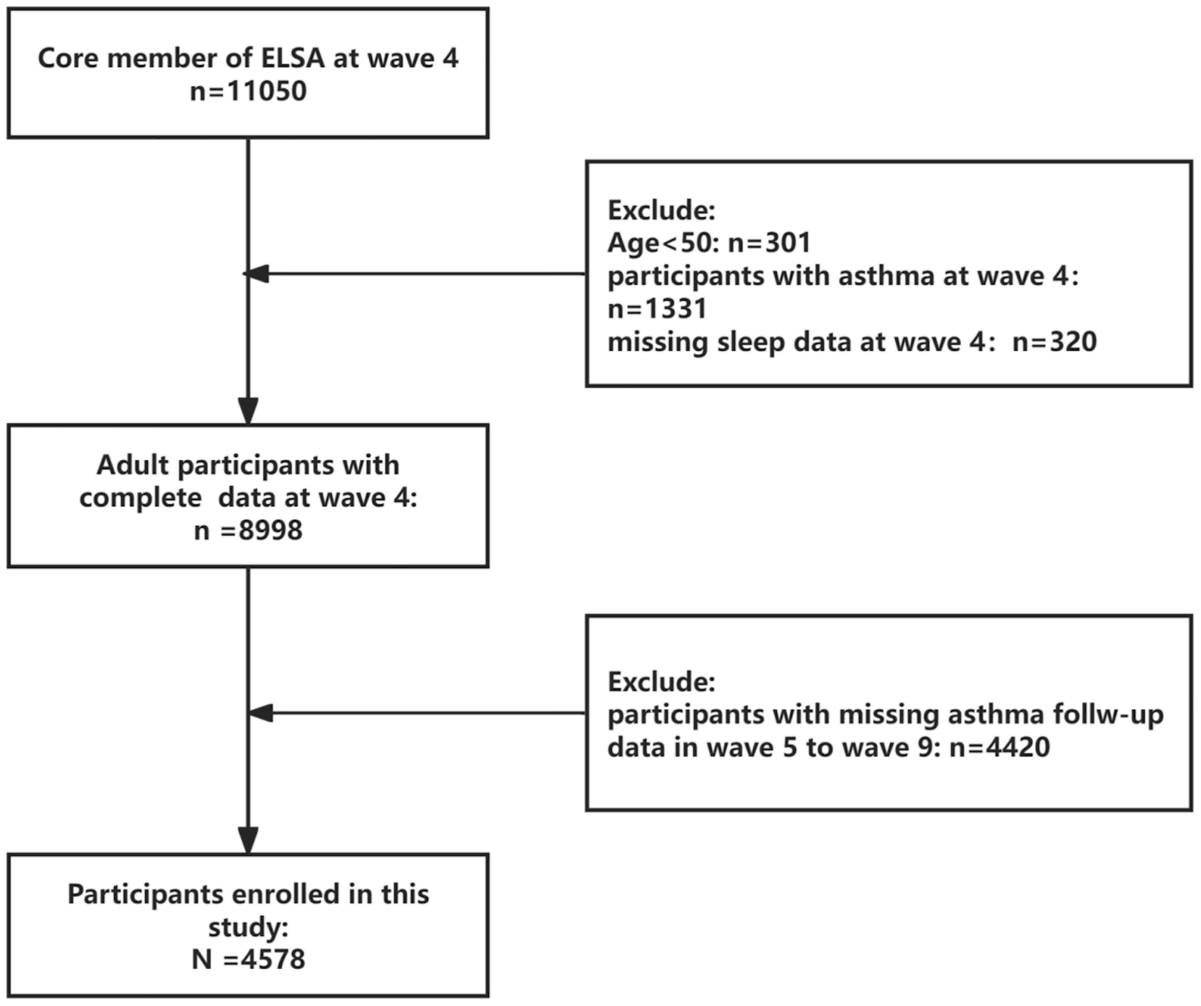
Research flowchart.

This investigation employed a prospective cohort design utilizing data from ELSA (37). ELSA constitutes a nationally representative longitudinal survey tracking community-dwelling adults aged ≥50 years, with repeated assessments of health status, socioeconomic indicators, and lifestyle behaviors across multidisciplinary domains. The present analysis established Wave 4 (2008–2009) as baseline, enrolling participants without prevalent asthma at study entry. Prospective follow-up extended through Wave 9 (2018–2019). From an initial pool of 11,050 Wave 4 participants, we excluded individuals with: (1) age < 50 (*n* = 301), (2) Pre-existing asthma diagnosis (*n* = 1,331), (3) Missing sleep data (*n* = 320), (4) Lost to follow-up before Wave 9 (*n* = 4,420). Figure 1 details the participant selection workflow, including inclusion/exclusion criteria and attrition patterns.

### 2.2 Assessment of exposure variables

#### 2.2.1 Sleep quality

Sleep quality was assessed using a modified Jenkins Sleep Scale (38). The scale includes four items: difficulty falling asleep, frequency of nocturnal awakenings, morning fatigue, and overall self-reported sleep quality. The total score ranges from 4 to 16, with higher scores indicating poorer sleep quality. Based on the total score, sleep quality was categorized into three groups: high quality (4–7 points), moderate quality (8–11 points), and low quality (≥12 points) (38, 39).

#### 2.2.2 Sleep duration

Sleep duration was assessed by self-reported average daily sleep duration (in hours).

### 2.3 Outcome

The primary outcome was the incidence of new asthma events during the 10-year follow-up period. New asthma diagnosis was determined using the core question from each follow-up wave: “Since your last interview, has a doctor ever told you that you have asthma?” Only participants who reported their first doctor-diagnosed asthma after baseline were considered to have experienced the outcome event.

### 2.4 Covariates

A range of potential confounders that could influence the relationship between sleep and asthma were also collected, including participants’ age, sex (Female/Male), race (Non-white/White), education level (Below high school/College or above/High school/Other), body mass index (BMI), smoking status (No/Yes), alcohol consumption (No/Yes), physical activity (at least one moderate or vigorous exercise per week: High/Low), income (annual total income of the family), and chronic diseases (e.g., hypertension, diabetes). To maintain data integrity, missing data were handled using multiple imputation.

### 2.5 Statistical analysis

Baseline characteristics of the study population were described according to sleep quality groups (Good, intermediate, poor). Continuous variables were expressed as means ± standard deviations or medians (interquartile ranges), while categorical variables were expressed as frequencies (percentages). Multivariable Cox proportional hazards regression models were used to assess the associations between baseline sleep quality (as a categorical variable with the high-quality group as the reference) and baseline sleep duration (as a continuous variable) with the risk of new-onset asthma during follow-up.

Model construction:

Model 1 (Unadjusted Model): Includes only sleep quality (or sleep duration).

Model 2 (Adjusted Model): Adjusts for demographic variables (age, sex, marital status, education, and race).

Model 3 (Fully Adjusted Model): Further adjusts for all selected potential confounders (BMI, smoking, alcohol consumption, physical activity level, depression, hypertension, diabetes, and hyperlipidemia).

Hazard ratios (HR) and their 95% confidence intervals (95% CI) were used to indicate the strength of the associations. For the sleep quality grouping analysis, the HR for the moderate and low-quality groups relative to the high-quality group was calculated. To test for a dose–response relationship between sleep quality and asthma risk, sleep quality was treated as an ordered variable in the Cox model, and its HR and P for trend value were computed. All statistical analyses were performed using R version 4.4.3. A two-sided test was used, with *p*-values < 0.05 considered statistically significant.

## 3 Results

### 3.1 Baseline characteristics

A total of 4,578 participants were included in this study, with a mean age of 63.10 (±7.78) years, of which 44.95% were male. Among the participants, 1785 individuals (38.99%) reported good sleep quality, 1907 (41.66%) reported moderate sleep quality, and 886 (19.35%) reported poor sleep quality (Table 1).

**Table 1:** Baseline characteristics of study populations across sleep quality.

Variable	Levels	*N*	Overall	Good	Intermediate	Poor	*p*-value
			*N* = 4,578	*N* = 1,785	*N* = 1,907	*N* = 886	
Age, year (sd)		4,578	63.10 (7.78)	63.02 (7.85)	63.56 (7.74)	62.26 (7.66)	<0.001
Sex, *n* (*p*%)		4,578					<0.001
	Female		2520.00 (55.05%)	837.00 (46.89%)	1071.00 (56.16%)	612.00 (69.07%)	
	Male		2058.00 (44.95%)	948.00 (53.11%)	836.00 (43.84%)	274.00 (30.93%)	
Race, *n* (*p*%)		4,578					0.01
	Non-white		128.00 (2.80%)	65.00 (3.64%)	37.00 (1.94%)	26.00 (2.93%)	
	White		4450.00 (97.20%)	1720.00 (96.36%)	1870.00 (98.06%)	860.00 (97.07%)	
Marital, *n* (*p*%)		4,578					<0.001
	Married or partnered		3399.00 (74.25%)	1375.00 (77.03%)	1436.00 (75.30%)	588.00 (66.37%)	
	Never married		235.00 (5.13%)	89.00 (4.99%)	88.00 (4.61%)	58.00 (6.55%)	
	Separated/divorced/widowed		944.00 (20.62%)	321.00 (17.98%)	383.00 (20.08%)	240.00 (27.09%)	
Education, *n* (*p*%)		4,578					<0.001
	Below high school		1262.00 (27.57%)	461.00 (25.83%)	513.00 (26.90%)	288.00 (32.51%)	
	College or above		1994.00 (43.56%)	854.00 (47.84%)	823.00 (43.16%)	317.00 (35.78%)	
	High school		973.00 (21.25%)	351.00 (19.66%)	425.00 (22.29%)	197.00 (22.23%)	
	Other		349.00 (7.62%)	119.00 (6.67%)	146.00 (7.66%)	84.00 (9.48%)	
Income (5th quintile), *n* (*p*%)		4,578					<0.001
	Q1		916.00 (20.01%)	365.00 (20.45%)	360.00 (18.88%)	191.00 (21.56%)	
	Q2		915.00 (19.99%)	308.00 (17.25%)	389.00 (20.40%)	218.00 (24.60%)	
	Q3		916.00 (20.01%)	335.00 (18.77%)	393.00 (20.61%)	188.00 (21.22%)	
	Q4		915.00 (19.99%)	369.00 (20.67%)	372.00 (19.51%)	174.00 (19.64%)	
	Q5		916.00 (20.01%)	408.00 (22.86%)	393.00 (20.61%)	115.00 (12.98%)	
BMI, mean (sd)		4,578	28.26 (5.08)	27.90 (4.85)	28.31 (5.03)	28.86 (5.57)	<0.001
Sleep duration, hour (sd)		4,578	6.86 (1.21)	7.26 (0.94)	6.96 (1.07)	5.86 (1.42)	<0.001
Depression, *n* (*p*%)		4,578					<0.001
	No		4078.00 (89.08%)	1729.00 (96.86%)	1749.00 (91.71%)	600.00 (67.72%)	
	Yes		500.00 (10.92%)	56.00 (3.14%)	158.00 (8.29%)	286.00 (32.28%)	
Hyperlipidemia, *n* (*p*%)		4,578					<0.001
	No		3291.00 (71.89%)	1360.00 (76.19%)	1345.00 (70.53%)	586.00 (66.14%)	
	Yes		1287.00 (28.11%)	425.00 (23.81%)	562.00 (29.47%)	300.00 (33.86%)	
Hypertension, *n* (*p*%)		4,578					0.07
	No		2408.00 (52.60%)	974.00 (54.57%)	991.00 (51.97%)	443.00 (50.00%)	
	Yes		2170.00 (47.40%)	811.00 (45.43%)	916.00 (48.03%)	443.00 (50.00%)	
Diabetes, *n* (*p*%)		4,578					0.04
	No		4264.00 (93.14%)	1677.00 (93.95%)	1778.00 (93.24%)	809.00 (91.31%)	
	Yes		314.00 (6.86%)	108.00 (6.05%)	129.00 (6.76%)	77.00 (8.69%)	
Physical activity, *n* (*p*%)		4,578					<0.001
	High		3881.00 (84.78%)	1558.00 (87.28%)	1653.00 (86.68%)	670.00 (75.62%)	
	Low		697.00 (15.22%)	227.00 (12.72%)	254.00 (13.32%)	216.00 (24.38%)	
Smoke, *n* (*p*%)		4,578					0.64
	No		2614.00 (57.10%)	1026.00 (57.48%)	1074.00 (56.32%)	514.00 (58.01%)	
	Yes		1964.00 (42.90%)	759.00 (42.52%)	833.00 (43.68%)	372.00 (41.99%)	
Drink, *n* (*p*%)		4,578					<0.001
	No		4162.00 (90.91%)	1648.00 (92.32%)	1751.00 (91.82%)	763.00 (86.12%)	
	Yes		416.00 (9.09%)	137.00 (7.68%)	156.00 (8.18%)	123.00 (13.88%)	
Follow-up time, month (sd)		4,578	119.22 (11.27)	119.81 (9.34)	119.03 (11.56)	118.48 (13.83)	0.530
Asthma, *n* (*p*%)		4,578					<0.001
	0		4422.00 (96.59%)	1746.00 (97.82%)	1837.00 (96.33%)	839.00 (94.70%)	
	1		156.00 (3.41%)	39.00 (2.18%)	70.00 (3.67%)	47.00 (5.30%)	

### 3.2 Associations between sleep duration, sleep quality, and asthma

Initially, no association between sleep duration and asthma risk was observed in any of the models (*p* > 0.05), as shown in Table 2. Regarding sleep quality, when analyzed as a continuous variable, a positive significant association with asthma was observed in all models, both unadjusted, partially adjusted, and fully adjusted. The hazard ratios (HRs) and 95% confidence intervals (CIs) were 1.10 (1.05–1.16), 1.09 (1.04–1.15), and 1.06 (1.01–1.12), respectively (Table 2). A similar trend was observed when sleep quality was analyzed as a categorical variable. Specifically, when fully adjusted, the risk of asthma for individuals with low and moderate sleep quality was 84 and 63% higher, respectively, compared to those with high sleep quality. A significant trend effect was also observed (*p* < 0.001). Furthermore, to test whether sleep quality influences asthma independently of sleep duration, we further adjusted for sleep duration in Model 3. The results still showed a significant association between sleep quality and asthma, with individuals with poor and moderate sleep quality having 108% (HR = 2.08, 95% CI: 1.25–3.46) and 66% (HR = 1.66, 95% CI: 1.11–2.48) higher risks of asthma compared to those with high sleep quality.

**Table 2:** Correlation between sleep quality, sleep duration and the risk of asthma.

Variables	Model 1		Model 2		Model 3	
	HR (95%CI)	*P*	HR (95%CI)	*P*	HR (95%CI)	*P*
Sleep quality scores	1.10 (1.05 ~ 1.16)	**<0.001**	1.09 (1.04 ~ 1.15)	**<0.001**	1.06 (1.01 ~ 1.12)	**0.019**
Sleep quality						
Good	1.00 (Reference)		1.00 (Reference)		1.00 (Reference)	
Intermediate	1.69 (1.14 ~ 2.49)	**0.009**	1.69 (1.14 ~ 2.50)	**0.009**	1.63 (1.09 ~ 2.42)	**0.016**
Poor	2.44 (1.59 ~ 3.73)	**<0.001**	2.28 (1.48 ~ 3.52)	**<0.001**	1.84 (1.16 ~ 2.92)	**0.010**
P for trend		**<0.001**		**<0.001**		**<0.001**
Sleep hours	0.97 (0.85 ~ 1.10)	0.644	0.99 (0.88 ~ 1.13)	0.935	1.05 (0.93 ~ 1.19)	0.454

HR, Hazard Ratio; CI, confidence interval.

Model 1: Crude.

Model 2: Adjust: race, sex, education, marital, age, income.

Model 3: Adjust: race, hyperlipidemia, hypertension, diabetes, physical activity, sex, education, marital, smoke, drink, age, BMI, depression.

The bolded values indicate that the *P*-values are less than 0.05.

### 3.3 Subgroup analyses

**Figure 2:**
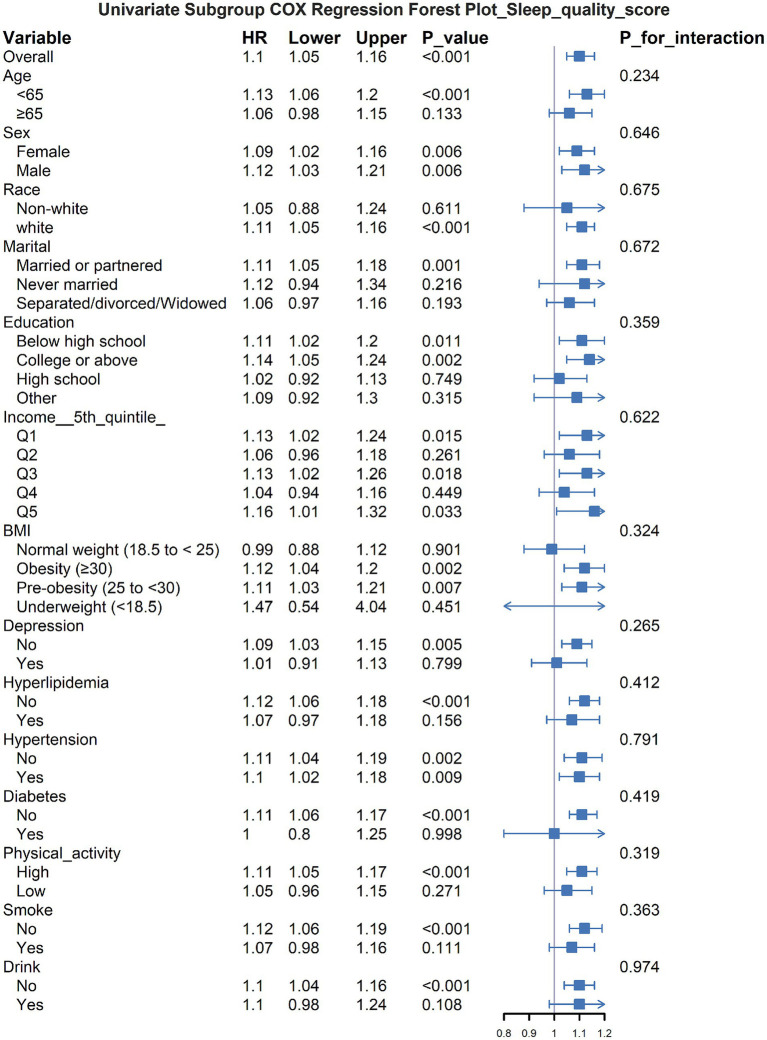
THE association between sleep quality and asthma in different subgroups.

The subgroup analysis revealed that the relationship between poor sleep quality and increased asthma risk varied across different groups (Figure 2). Specifically, the association was more significant in younger individuals, females, white participants, those with a partner, and those with higher educational levels. Additionally, in populations with obesity, hyperlipidemia, hypertension, and other health problems, poor sleep quality appeared to have a stronger impact on asthma risk. No significant interaction effects were observed (all P_interaction > 0.05), indicating that the influence of sleep quality on asthma risk was relatively consistent across different subgroups.

## 4 Discussion

This investigation represents the first large-scale prospective cohort analysis examining longitudinal associations between sleep characteristics (quality and duration) and incident asthma risk among adults aged ≥50 years, utilizing data from the English Longitudinal Study of Ageing (ELSA). Following comprehensive adjustment for pertinent covariates—including demographic factors (age, sex), behavioral parameters (smoking status, physical activity), socioeconomic indicators, and comorbid conditions—baseline sleep quality demonstrated a significant dose-dependent relationship with 10-year asthma incidence. Critically, no significant association emerged between nocturnal sleep duration and asthma risk. This result highlights the potential independent and important role of sleep quality, rather than just sleep duration, in influencing the risk of asthma in middle-aged and older adults.

Older adults constitute a high-risk population for both asthma and sleep-related disorders. With advancing age, structural and rhythmic alterations in sleep patterns emerge, accompanied by an elevated prevalence of insomnia and diminished sleep quality (40). Similarly, the incidence of asthma among older adults warrants considerable attention, given its distinct clinical manifestations, diagnostic challenges, and therapeutic considerations. Sleep disturbances, as a prevalent public health concern, have received increasing scrutiny due to their substantial implications for overall health (41, 42). Critically, poor sleep quality directly impairs multidimensional quality of life (QoL), reducing physical vitality and cognitive function (43). From a life hygiene perspective, maintaining optimal sleep is fundamental for immune-metabolic homeostasis (44). Consistent with prior research, our findings demonstrate a significant association between impaired sleep quality and heightened asthma risk (15, 20, 24). One study revealed that poor sleep quality not only correlated with increased asthma incidence but also exhibited an interactive effect with depression, further exacerbating asthma susceptibility (20). Additionally, evidence indicates that sleep-related breathing disorders (SRBD) serve as an independent risk factor for suboptimal asthma control in pediatric populations, adversely affecting pulmonary function (24). Among urban children, nocturnal asthma symptoms were linked to diminished sleep quality and a concomitant decline in parental quality of life (15). Additionally, existing literature has predominantly examined the influence of sleep disturbances on asthma management, symptom severity, and patient well-being (45–47). For instance, a cross-sectional investigation of asthma patients demonstrated that over 50% experienced sleep disorders, with sleep quality significantly correlated with both asthma control and quality of life (15). Another longitudinal study observed that sleep impairments in asthmatic children were associated with progressive deterioration in health-related quality of life, wherein the relationship between asthma control status and asthma-specific quality of life was mediated by nocturnal sleep quality and daytime somnolence (46). A recent meta-analysis systematically evaluated the association between sleep disorders—including insomnia, poor sleep quality, and sleep deprivation—and asthma, concluding that such disturbances were linked to an elevated asthma risk (48). However, the majority of these studies have either focused on individuals with pre-existing asthma diagnoses or employed cross-sectional methodologies, thereby limiting the ability to establish a definitive temporal or causal relationship between sleep disturbances and asthma onset. In contrast, the present study utilizes a prospective cohort design and excludes baseline asthma cases, thereby providing more robust evidence for the predictive role of sleep quality in the development of incident asthma.

Regarding the relationship between sleep duration and asthma, this study did not find a significant association, which contrasts with the results of some other studies. For example, many studies have shown that both short and long sleep durations are associated with an increased asthma risk (28–30, 49, 50). However, our findings are still supported by some studies indicating that sleep duration is not related to asthma risk (26, 27, 36). This inconsistency may arise from the fact that self-reported sleep duration may not accurately capture actual sleep efficiency or structure. The impact of sleep duration may be modulated by age, underlying health conditions, or specific asthma phenotypes, and its independent effect may not be as significant as that of sleep quality. Our study focused on middle-aged and older adults, whose sleep needs, patterns, and comorbidity profiles differ from those of children and adolescents. The negative result from this study emphasizes that in asthma primary prevention strategies, focusing on and improving sleep quality may be more important than solely pursuing a specific sleep duration. This aligns with some studies that stress the central role of sleep quality in overall health.

The potential mechanisms through which poor sleep quality increases asthma risk are multifactorial and may involve immune-inflammatory responses, neuroendocrine dysregulation, and autonomic nervous system dysfunction. First, sleep is crucial for maintaining normal immune function (51). Disrupted sleep or sleep deprivation can elevate levels of pro-inflammatory cytokines (such as IL-6, TNF-α, and C-reactive protein) while suppressing the production of anti-inflammatory cytokines, thereby exacerbating systemic inflammation (52–55). Given that asthma is fundamentally a chronic inflammatory disease of the airways, the inflammatory imbalance induced by poor sleep quality may facilitate the onset of airway inflammation and heighten asthma susceptibility. Second, sleep disturbances can lead to endocrine and metabolic dysregulation, including abnormalities in glucose metabolism, hormonal fluctuations, and the development of insulin resistance, which collectively contribute to the onset of asthma (56–60). Critically, these metabolic alterations interact with respiratory and systemic conditions: Hyperinsulinemia exacerbates obstructive sleep apnea (OSA) by increasing upper airway collapsibility, while OSA-related nocturnal hypoxia further worsens insulin resistance via HIF-1α activation, creating a vicious cycle that amplifies asthma risk (61). Concurrently, insulin resistance elevates pro-inflammatory cytokines that sensitize trigeminal nociceptors, explaining higher prevalence of temporomandibular disorders (TMD) in asthmatics with poor sleep (62). Furthermore, the autonomic nervous system plays a critical role in regulating airway tone and inflammation. Sleep disorders may provoke excessive activation of the sympathetic nervous system, coupled with a relative insufficiency of parasympathetic nervous system activity, thus enhancing bronchoconstriction and mucus production, which can trigger or exacerbate asthma symptoms (63). Additionally, sleep disorders are frequently associated with negative emotional states such as anxiety and depression (64, 65), both of which are recognized as risk factors or triggers for asthma (66, 67). Therefore, poor sleep quality may indirectly elevate asthma risk by mediating psychological stress responses. Finally, specific sleep disorders, such as obstructive sleep apnea (OSA), have been identified as both risk factors and comorbidities for asthma (68). While this study assessed overall sleep quality, it is possible that the effects of specific sleep disorders, such as OSA, were also captured, despite not being explicitly identified.

This study has several limitations. First, both sleep quality and duration relied on self-reported measures, introducing potential recall and reporting biases. Although the questionnaire demonstrated good reliability and validity (38, 39, 69), it remains inferior to objective monitoring methods (e.g., polysomnography, actigraphy). Future research should incorporate objective sleep parameters. Second, asthma diagnosis was based on self-reported physician confirmation, common in large epidemiology but susceptible to misclassification. Inclusion of objective measures (e.g., lung function tests, airway inflammation markers) would enhance diagnostic accuracy. Third, despite adjustment for known confounders, residual confounding (e.g., unmeasured environmental exposures like air pollution/allergens, early-life factors, or specific mental health conditions) remains possible. Fourth, the findings from participants aged ≥50 years may not generalize to younger populations (e.g., children, adolescents), whose asthma phenotypes and sleep patterns differ. Validation in diverse age and ethnic groups is warranted. Fifth, focusing solely on overall sleep quality precluded assessment of differential impacts by specific sleep disorder types on asthma risk.

Despite these limitations, the findings have significant public health implications. Given the high prevalence of poor sleep quality among middle-aged and older adults (40) and the substantial burden of asthma on quality of life and healthcare systems (2), this study suggests that enhancing sleep quality may represent a novel preventive target for new-onset asthma in this population. Clinicians should routinely assess sleep quality during health evaluations of middle-aged and older adults. For individuals with poor sleep, prompt identification of underlying causes and implementation of targeted interventions—such as cognitive behavioral therapy for insomnia (CBT-I), sleep hygiene optimization, appropriate physical activity, or management of comorbid physical/mental health conditions—are essential. Concurrently, public health initiatives should strengthen sleep health promotion to raise awareness of its importance.

## 5 Conclusion

In conclusion, the findings of this large-scale prospective cohort study suggest that poor sleep quality is an independent risk factor for new-onset asthma in middle-aged and older adults, and this association is independent of sleep duration. This finding provides a new perspective on asthma primary prevention, indicating that improving sleep quality could be a potential public health strategy to reduce the risk of asthma in this population. Future research should further explore the underlying mechanisms, validate these findings in different populations, and assess the effectiveness of interventions aimed at improving sleep quality.

## Data Availability

The original contributions presented in the study are included in the article/supplementary material, further inquiries can be directed to the corresponding author.

## References

[1] Bousquet J Khaltaev N Cruz AA Denburg J Fokkens WJ Togias A. Allergic rhinitis and its impact on asthma (ARIA) 2008 update (in collaboration with the World Health Organization, GA(2)LEN and AllerGen). Allergy. (2008) 63:8–160. doi: 10.1111/j.1398-9995.2007.01620.x, PMID: 18331513

[2] Wang Z Li Y Gao Y Fu Y Lin J Lei X. Global, regional, and national burden of asthma and its attributable risk factors from 1990 to 2019: a systematic analysis for the global burden of disease study 2019. Respir Res. (2023) 24:169. doi: 10.1186/s12931-023-02475-6, PMID: 37353829 PMC10288698

[3] Cao Y Chen S Chen X Zou W Liu Z Wu Y. Global trends in the incidence and mortality of asthma from 1990 to 2019: an age-period-cohort analysis using the global burden of disease study 2019. Front Public Health. (2022) 10:1036674. doi: 10.3389/fpubh.2022.1036674, PMID: 36483262 PMC9723391

[4] Tay TR Radhakrishna N Hore-Lacy F Smith C Hoy R Dabscheck E. Comorbidities in difficult asthma are independent risk factors for frequent exacerbations, poor control and diminished quality of life. Respirology. (2016) 21:1384–90. doi: 10.1111/resp.12838, PMID: 27363539

[5] Sun H Du Z Yu H Hu C Du Y Qin Y. Excessive daytime sleepiness is associated with increased residual cardiovascular risks among coronary artery disease patients with obstructive sleep apnea. Sleep Med. (2024) 115:131–6. doi: 10.1016/j.sleep.2024.02.004, PMID: 38359592

[6] Clark AJ Salo P Lange T Jennum P Virtanen M Pentti J. Onset of impaired sleep and cardiovascular disease risk factors: a longitudinal study. Sleep. (2016) 39:1709–18. doi: 10.5665/sleep.6098, PMID: 27397560 PMC4989260

[7] Deacon-Crouch M Begg S Skinner T. Is sleep duration associated with overweight/obesity in indigenous australian adults? BMC Public Health. (2020) 20:1229. doi: 10.1186/s12889-020-09287-z, PMID: 32787811 PMC7424988

[8] Martynowicz H Wichniak A Więckiewicz M. Sleep disorders and cardiovascular risk: focusing on sleep fragmentation. Dent Med Probl. (2024) 61:475–7. doi: 10.17219/dmp/185395, PMID: 38517218

[9] Martynowicz H Michalek-Zrabkowska M Gac P Blaszczyk B Fulek M Frosztega W. Performance evaluation of portable respiratory polygraphy for assessing sleep bruxism in adults. J Oral Rehabil. (2024) 51:1862–71. doi: 10.1111/joor.13733, PMID: 38751053

[10] Orzeszek S Martynowicz H Smardz J Wojakowska A Bombała W Mazur G. Assessment of sleep quality in patients with orofacial pain and headache complaints: a polysomnographic study. Dent Med Probl. (2024) 61:549–62. doi: 10.17219/dmp/177008, PMID: 38832763

[11] Abdul Razak MR Chirakalwasan N. Obstructive sleep apnea and asthma. Asian Pac J Allergy Immunol. (2016) 34:265–71. doi: 10.12932/AP0828, PMID: 28042927

[12] Doenges J Kuckuck E Cassel W Hildebrandt O Weissflog A Sohrabi K. Disease control in patients with asthma and respiratory symptoms (wheezing, cough) during sleep. Asthma Res Pract. (2020) 6:9. doi: 10.1186/s40733-020-00062-w, PMID: 32983550 PMC7513478

[13] Tinschert P Rassouli F Barata F Steurer-Stey C Fleisch E Puhan MA. Nocturnal cough and sleep quality to assess asthma control and predict attacks. J Asthma Allergy. (2020) 13:669–78. doi: 10.2147/JAA.S278155, PMID: 33363391 PMC7754262

[14] Scheer FAJL Hilton MF Evoniuk HL Shiels SA Malhotra A Sugarbaker R. The endogenous circadian system worsens asthma at night independent of sleep and other daily behavioral or environmental cycles. Proc Natl Acad Sci USA. (2021) 118:e2018486118. doi: 10.1073/pnas.2018486118, PMID: 34493686 PMC8449316

[15] Fagnano M Bayer AL Isensee CA Hernandez T Halterman JS. Nocturnal asthma symptoms and poor sleep quality among urban school children with asthma. Acad Pediatr. (2011) 11:493–9. doi: 10.1016/j.acap.2011.05.006, PMID: 21816697 PMC3481184

[16] Min YZ Subbarao P Narang I. The bidirectional relationship between asthma and obstructive sleep apnea: which came first? J Pediatr. (2016) 176:10–6. doi: 10.1016/j.jpeds.2016.05.058, PMID: 27318377

[17] Hu Z Song X Hu K. The effect of short sleep duration on the development of asthma. Int J Clin Pract. (2022) 2022:1–7. doi: 10.1155/2022/3378821, PMID: 35685599 PMC9159162

[18] Imani MM Sadeghi M Khazaie H Emami M Sadeghi Bahmani D Brand S. Evaluation of serum and plasma interleukin-6 levels in obstructive sleep apnea syndrome: a meta-analysis and meta-regression. Front Immunol. (2020) 11:1343. doi: 10.3389/fimmu.2020.01343, PMID: 32793188 PMC7385225

[19] Petrov KK Hayley A Catchlove S Savage K Stough C. Is poor self-rated sleep quality associated with elevated systemic inflammation in healthy older adults? Mech Ageing Dev. (2020) 192:111388. doi: 10.1016/j.mad.2020.111388, PMID: 33080282

[20] Lai Y Zhang X Dong H Li M. The interaction effects between depression and sleep status on asthma: a national cross-sectional study. Front Psych. (2024) 15:1487550. doi: 10.3389/fpsyt.2024.1487550, PMID: 39479594 PMC11521870

[21] Liu Y Croft JB Wheaton AG Perry GS Chapman DP Strine TW. Association between perceived insufficient sleep, frequent mental distress, obesity and chronic diseases among US adults, 2009 behavioral risk factor surveillance system. BMC Public Health. (2013) 13:84. doi: 10.1186/1471-2458-13-84, PMID: 23360346 PMC3562519

[22] Zhang J Lam SP Li SX Yu MWM Li AM Ma RCW. Long-term outcomes and predictors of chronic insomnia: a prospective study in Hong Kong Chinese adults. Sleep Med. (2012) 13:455–62. doi: 10.1016/j.sleep.2011.11.015, PMID: 22425578

[23] Brumpton B Mai X-M Langhammer A Laugsand LE Janszky I Strand LB. Prospective study of insomnia and incident asthma in adults: the HUNT study. Eur Respir J. (2017) 49:1601327. doi: 10.1183/13993003.01327-2016, PMID: 28153868

[24] Sheen YH Choi SH Jang SJ Baek JH Jee HM Kim MA. Poor sleep quality has an adverse effect on childhood asthma control and lung function measures. Pediatr Int. (2017) 59:917–22. doi: 10.1111/ped.13312 28452099

[25] Tinschert P Rassouli F Barata F Steurer-Stey C Fleisch E Puhan MA. Prevalence of nocturnal cough in asthma and its potential as a marker for asthma control (MAC) in combination with sleep quality: protocol of a smartphone-based, multicentre, longitudinal observational study with two stages. BMJ Open. (2019) 9:e026323. doi: 10.1136/bmjopen-2018-026323, PMID: 30617104 PMC6326321

[26] Seow LSE Tan XW Chong SA Vaingankar JA Abdin E Shafie S. Independent and combined associations of sleep duration and sleep quality with common physical and mental disorders: results from a multi-ethnic population-based study. PLoS One. (2020) 15:e0235816. doi: 10.1371/journal.pone.0235816, PMID: 32673344 PMC7365445

[27] Chen Y Yang Q Zhao K Wu Z Shen X Li S. Associations of sleep characteristics with atopic disease: a cross-sectional study among Chinese adolescents. Allergy Asthma Clin Immunol. (2021) 17:21. doi: 10.1186/s13223-021-00516-7, PMID: 33618771 PMC7898413

[28] Hu Z Song X Hu K Ruan Y Zeng F. Association between sleep duration and asthma in different weight statuses (CHNS 2009-2015). Sleep Breath. (2020) 25:493–502. doi: 10.1007/s11325-020-02081-6, PMID: 32335852

[29] Choi JH Nam GE Kim DH Lee JY Han KD Cho JH. Association between sleep duration and the prevalence of atopic dermatitis and asthma in young adults. Asian Pac J Allergy Immunol. (2016) 35:150–5. doi: 10.12932/AP0772 27996279

[30] Han CH Chung JH. Association of asthma and sleep insufficiency among south korean adolescents: analysis of web-based self-reported data from the korean youth risk behavior web-based survey. J Asthma. (2020) 57:253–61. doi: 10.1080/02770903.2019.1565827, PMID: 30657005

[31] Lim MS Lee CH Sim S Hong SK Choi HG. Physical activity, sedentary habits, sleep, and obesity are associated with asthma, allergic rhinitis, and atopic dermatitis in korean adolescents. Yonsei Med J. (2017) 58:1040–6. doi: 10.3349/ymj.2017.58.5.1040, PMID: 28792151 PMC5552632

[32] Nutakor JA Dai B Gavu AK Antwi O-A. Relationship between chronic diseases and sleep duration among older adults in Ghana. Qual Life Res. (2020) 29:2101–10. doi: 10.1007/s11136-020-02450-4, PMID: 32100183

[33] Yang G Han Y-Y Sun T Li L Rosser F Forno E. Sleep duration, current asthma, and lung function in a nationwide study of U.S. adults. Am J Respir Crit Care Med. (2019) 200:926–9. doi: 10.1164/rccm.201905-1004LE, PMID: 31225970 PMC6812440

[34] Bakour C O’Rourke K Schwartz S Wang W Sappenfield W Couluris M. Sleep duration, obesity, and asthma, in florida adolescents: analysis of data from the florida youth risk behavior survey (2009-2013). Sleep Breath. (2017) 21:1039–45. doi: 10.1007/s11325-017-1460-2, PMID: 28093685

[35] Dashti HS Redline S Saxena R. Polygenic risk score identifies associations between sleep duration and diseases determined from an electronic medical record biobank. Sleep. (2019) 42:zsy247. doi: 10.1093/sleep/zsy247, PMID: 30521049 PMC6424085

[36] Ma Y Tang J Wen Y Hu Y Liang J Jiang L. Associations of sleep problems with asthma and allergic rhinitis among Chinese preschoolers. Sci Rep. (2022) 12:8102. doi: 10.1038/s41598-022-12207-3, PMID: 35577978 PMC9110737

[37] Steptoe A Breeze E Banks J Nazroo J. Cohort profile: the English longitudinal study of ageing. Int J Epidemiol. (2012) 42:1640–8. doi: 10.1093/ije/dys168, PMID: 23143611 PMC3900867

[38] Jenkins CD Stanton B-A Niemcryk SJ Rose RM. A scale for the estimation of sleep problems in clinical research. J Clin Epidemiol. (1988) 41:313–21. doi: 10.1016/0895-4356(88)90138-2, PMID: 3351539

[39] Song Y Chang Z Song C Cui K Yuan S Qiao Z. Association of sleep quality, its change and sleep duration with the risk of type 2 diabetes mellitus: findings from the English longitudinal study of ageing. Diabetes Metab Res Rev. (2023) 39:e3669. doi: 10.1002/dmrr.3669, PMID: 37288700

[40] Wang X Wang R Zhang D. Bidirectional associations between sleep quality/duration and multimorbidity in middle-aged and older people Chinese adults: a longitudinal study. BMC Public Health. (2024) 24:708. doi: 10.1186/s12889-024-17954-8, PMID: 38443848 PMC10916205

[41] Ohayon M Wickwire EM Hirshkowitz M Albert SM Avidan A Daly FJ. National sleep foundation’s sleep quality recommendations: first report. Sleep Health. (2017) 3:6–19. doi: 10.1016/j.sleh.2016.11.006, PMID: 28346153

[42] Sletten TL Weaver MD Foster RG Gozal D Klerman EB Rajaratnam SMW. The importance of sleep regularity: a consensus statement of the national sleep foundation sleep timing and variability panel. Sleep Health. (2023) 9:801–20. doi: 10.1016/j.sleh.2023.07.016, PMID: 37684151

[43] Chattu VK Manzar MD Kumary S Burman D Spence DW Pandi-Perumal SR. The global problem of insufficient sleep and its serious public health implications. Health Care (Don Mills). (2018) 7:1. doi: 10.3390/healthcare7010001, PMID: 30577441 PMC6473877

[44] Faraut B Boudjeltia KZ Vanhamme L Kerkhofs M. Immune, inflammatory and cardiovascular consequences of sleep restriction and recovery. Sleep Med Rev. (2012) 16:137–49. doi: 10.1016/j.smrv.2011.05.001, PMID: 21835655

[45] Braido F Baiardini I Ferrando M Scichilone N Santus P Petrone A. The prevalence of sleep impairments and predictors of sleep quality among patients with asthma. J Asthma. (2020) 58:481–7. doi: 10.1080/02770903.2019.1711391, PMID: 31903810

[46] Li Z Thompson LA Gross HE Shenkman EA Reeve BB DeWalt DA. Longitudinal associations among asthma control, sleep problems, and health-related quality of life in children with asthma: a report from the PROMIS(®) pediatric asthma study. Sleep Med. (2016) 20:41–50. doi: 10.1016/j.sleep.2015.12.003, PMID: 27318225 PMC4913028

[47] Garden M O’Callaghan M Suresh S Mamum AA Najman JM. Asthma and sleep disturbance in adolescents and young adults: a cohort study. J Paediatr Child Health. (2016) 52:1019–25. doi: 10.1111/jpc.13234 27288910

[48] Liu X Hong C Liu Z Fan L Yin M Chen Y. Association of sleep disorders with asthma: a meta-analysis. BMJ Open Respir Res. (2023) 10:e001661. doi: 10.1136/bmjresp-2023-001661, PMID: 37735102 PMC10514641

[49] Hu Z Tian Y Song X Hu K Yang A. Associations between incident asthma with comorbidity profiles, night sleep duration, and napping duration trajectories: a 7-year prospective study. Int J Public Health. (2022) 67:1604939. doi: 10.3389/ijph.2022.1604939, PMID: 35872705 PMC9305997

[50] Bakour C Schwartz SW Wang W Sappenfield WM Couluris M Chen H. Sleep duration patterns from adolescence to young adulthood and the risk of asthma. Ann Epidemiol. (2020) 49:20–6. doi: 10.1016/j.annepidem.2020.07.003 32681981

[51] Besedovsky L Lange T Haack M. The sleep-immune crosstalk in health and disease. Physiol Rev. (2019) 99:1325–80. doi: 10.1152/physrev.00010.2018, PMID: 30920354 PMC6689741

[52] Wang CY Wang J Zhang L Zhang SW Wang L Zhao SZ. Self-reported insufficient sleep is associated with clinical and inflammatory features of asthma: a prospective cohort study. J Allergy Clin Immunol Pract. (2023) 11:1200–1210.e4. doi: 10.1016/j.jaip.2022.12.011, PMID: 36581067

[53] Chennaoui M Gomez-Merino D Drogou C Geoffroy H Dispersyn G Langrume C. Effects of exercise on brain and peripheral inflammatory biomarkers induced by total sleep deprivation in rats. J Inflamm. (2015) 12:56. doi: 10.1186/s12950-015-0102-3, PMID: 26425116 PMC4588685

[54] Kang WS Park HJ Chung J-H Kim JW. REM sleep deprivation increases the expression of interleukin genes in mice hypothalamus. Neurosci Lett. (2013) 556:73–8. doi: 10.1016/j.neulet.2013.09.050, PMID: 24080377

[55] Axelsson J Rehman J Akerstedt T Ekman R Miller GE Höglund CO. Effects of sustained sleep restriction on mitogen-stimulated cytokines, chemokines and T helper 1/ T helper 2 balance in humans. PLoS One. (2013) 8:e82291. doi: 10.1371/journal.pone.0082291, PMID: 24349251 PMC3859577

[56] Van Cauter E. Sleep disturbances and insulin resistance. Diabet Med. (2011) 28:1455–62. doi: 10.1111/j.1464-5491.2011.03459.x 21950773

[57] Morgan D Tsai SC. Sleep and the endocrine system. Sleep Med Clin. (2016) 11:115–26. doi: 10.1016/j.jsmc.2015.10.002, PMID: 26972038

[58] Koren D O’Sullivan KL Mokhlesi B. Metabolic and glycemic sequelae of sleep disturbances in children and adults. Curr Diab Rep. (2014) 15:562. doi: 10.1007/s11892-014-0562-5, PMID: 25398202 PMC4467532

[59] Tesse R Schieck M Kabesch M. Asthma and endocrine disorders: shared mechanisms and genetic pleiotropy. Mol Cell Endocrinol. (2011) 333:103–11. doi: 10.1016/j.mce.2010.11.032, PMID: 21134413

[60] Malicka B Kaczmarek U Skośkiewicz-Malinowska K. Prevalence of xerostomia and the salivary flow rate in diabetic patients. Adv Clin Exp Med. (2014) 23:225–33. doi: 10.17219/acem/37067, PMID: 24913113

[61] Karuga FF Jaromirska J Sochal M Białasiewicz P Gabryelska A. Association between glucose metabolism, the circadian cycle and hypoxia: evaluation of the NPAS2 and rev-erb-α protein serum levels in obstructive sleep apnea patients - a pilot study. Dent Med Probl. (2024) 61:465–9. doi: 10.17219/dmp/185718, PMID: 38804230

[62] Więckiewicz M Lavigne G Martynowicz H. Decrypting the putative interrelation between sleep bruxism, masticatory muscle pain and sleep breathing disorders: nosology and the role of hypoxia. Dent Med Probl. (2024) 61:165–7. doi: 10.17219/dmp/175686, PMID: 38488764

[63] Özdemir F Boşnak Güçlü M Göktaş HE Oğuzülgen IK. Maximal exercise capacity, peripheral muscle strength, sleep quality, and quality of life in adult patients with stable asthma. J Asthma. (2024) 62:608–20. doi: 10.1080/02770903.2024.2425369, PMID: 39498583

[64] Pandi-Perumal SR Monti JM Burman D Karthikeyan R BaHammam AS Spence DW. Clarifying the role of sleep in depression: a narrative review. Psychiatry Res. (2020) 291:113239. doi: 10.1016/j.psychres.2020.113239, PMID: 32593854

[65] Zhai L Zhang H Zhang D. Sleep duration and depression among adults: a meta-analysis of prospective studies. Depress Anxiety. (2015) 32:664–70. doi: 10.1002/da.22386, PMID: 26047492

[66] Han Y-Y Yan Q Chen W Celedón JC. Child maltreatment, anxiety and depression, and asthma among british adults in the UK biobank. Eur Respir J. (2022) 60:2103160. doi: 10.1183/13993003.03160-2021, PMID: 35301250 PMC9481745

[67] Jiang M Qin P Yang X. Comorbidity between depression and asthma via immune-inflammatory pathways: a meta-analysis. J Affect Disord. (2014) 166:22–9. doi: 10.1016/j.jad.2014.04.027, PMID: 25012406

[68] Althoff MD Ghincea A Wood LG Holguin F Sharma S. Asthma and three colinear comorbidities: obesity, OSA, and GERD. J Allergy Clin Immunol Pract. (2021) 9:3877–84. doi: 10.1016/j.jaip.2021.09.003, PMID: 34506967 PMC8578370

[69] Yang P Tian L Xia Y Hu M Xiao X Leng Y. Association of sleep quality and its change with the risk of depression in middle-aged and elderly people: a 10-year cohort study from England. J Affect Disord. (2025) 373:245–52. doi: 10.1016/j.jad.2024.12.079, PMID: 39732401

